# Necrotising Otitis Externa: A Review of Imaging Modalities

**DOI:** 10.7759/cureus.20675

**Published:** 2021-12-25

**Authors:** Hammaad A Khan

**Affiliations:** 1 Otolaryngology - Head and Neck Surgery, Aintree University Hospital, Liverpool, GBR

**Keywords:** radionucleotide scan, indium in-111 labelled leukocyte scanning, gallium citrate ga-67 bone scan, technetium tc-99 bone scan, f-18 fdg pet/ct, ct scan of temporal bone, necrotising external otitis, nuclear medicine imaging, malignant otitis externa, skull base osteomyelitis

## Abstract

Necrotising Otitis Externa (NOE) has often posed some challenges in view of diagnosis and management by clinicians. One such challenge is the appropriate and timely use of imaging techniques since its use is critical not only in diagnosis but also in determining the extent and resolution of the disease. Hence, doctors in both primary and secondary health care need to be familiar with presenting symptoms while specialists need to be appraised of advances in imagining techniques in diagnosis and management of NOE. Whilst there is a general consensus amongst clinicians on some aspects of management of NOE, there is very limited consensus on the use of imaging modalities. There is no single modality of imaging that can provide a complete picture of diagnosis, disease progression and resolution. This review aims to highlight the strengths and weaknesses of various imaging techniques used in the diagnosis and management of NOE over the years and whether a multi-modal imaging technique at particular stages of the disease may provide better management outcomes.

## Introduction and background

Necrotising Otitis Externa (NOE), also referred to as Malignant External Otitis, is a rare but severe infection of the external auditory canal and surrounding structures. This disease commonly affects immuno-compromised and elderly persons with Diabetes Mellitus [1]. NOE has posed some challenges for clinicians in its diagnosis and management. One such challenge is the appropriate and timely use of imaging techniques, which is not only critical in diagnosis but also in determining the extent and resolution of the disease. Early diagnosis and prompt management by relevant medical teams are key in NOE, which has a high morbidity-mortality rate. Hence, doctors in both primary and secondary health care need to be familiar with presenting symptoms while specialists need to be appraised of advances in imagining techniques in the management of NOE. There is no single modality of imaging that can provide a complete picture of the diagnosis, disease progression and resolution of NOE [1]. Therefore, a multi-modal approach involving various imaging modalities at particular stages of the disease may provide better management outcomes. However, it is also important to note the use of these imaging modalities is also dependant on the availability of resources and other factors around the world.

What is necrotising otitis externa?

Necrotising otitis externa is a rare but severe and aggressive infection of the external auditory canal and surrounding structures. This commonly spreads to involve the periosteum and reaches the bone of the skull base. Once bone involvement is confirmed on radiological findings, it is also referred to as Skull Base Osteomyelitis (SBO). However, SBO is often referred to as a complication of NOE. As mentioned earlier NOE is also widely known as Malignant Otitis Externa as it mimics a neoplastic condition where the disease spreads and deteriorates rapidly like malignancy [1]. This is actually a misnomer as the condition is not in reality a neoplastic condition. It is known as NOE due to extensive soft tissue involvement. If untreated, cranial neuropathies of which the facial nerve is the most common can develop due to sub-temporal extension of the infection. Early diagnosis and prompt management by relevant medical teams are thus key in NOE, which has a high morbidity-mortality rate. Hence delays in the diagnosis of NOE can affect disease outcomes significantly [2].

On clinical examination, granulation tissue is present on the floor of the osteocartilaginous junction and otoscopic examination can reveal exposed bone [3]. Granulation is virtually pathognomonic of NOE except in those with HIV/AIDS where granulation tissue may be absent in the external auditory canal.

Infection begins in the skin and cartilage of the external auditory meatus and spreads rapidly and causes necrosis of the surrounding soft tissues, cartilage and bones by invading them and even spreads to the cranial nerves. NOE is referred to as 'necrotising and invasive' due to the fact the infection invades the adjacent peri-auricular tissues into the cartilage and bones resulting in necrosis and osteomyelitis of the temporal bone as well as the base of the skull [4].

Spread of the disease outside the external auditory canal occurs rapidly and progressively through the fissures of Santorini and the tympano-mastoid suture to the skull base. Periostitis spreads along the undersurface of the skull base to involve the stylo-mastoid foramen and then the jugular and hypoglossal foramina and hence the facial nerve which lies in close proximity here can be affected easily [5]. The disease can be fatal if treatment is not aggressive and timely, especially if it spreads outside the auditory canal with the involvement of the cranial nerves [4].

The symptoms of NOE are easily recognisable. These include persistent and foul-smelling yellow or green drainage from the ear (otorrhea) accompanied by otalgia that gets worse when moving the head and at night. Other symptoms include hearing loss, persistent itching in the ear canal, fever, difficulty in swallowing and weakness in the facial muscles. Laryngitis may be experienced by those where the infection travels to the larynx. In addition, the skin around the ear appears swollen and red [6].

Diagnosis

The diagnosis of NOE requires a high index of suspicion is mainly based on several clinical findings, markers such as an elevated erythrocyte sedimentation rate, presence of certain bacteriological pathogens found on laboratory analysis, radiographic evidence of soft tissue with or without bone erosion in the external canal and infra-temporal fossa [6]. A physical exam including a complete medical history to identify underlying conditions that may compromise the immune system is important. In addition, an ear examination may reveal granulation tissue or drainage which may indicate an infection. A sample is then sent for analysis and the identification of particular pathogens such as *Pseudomonas aeruginosa, *Methicillin-resistant *Staphylococcus aureus *(MRSA),* Staphylococcus epidermidis, Proteus, Klebsiella. Aspergillus fumigatus* and *Candida *species can indicate NOE [6]. Additional tests such as a neurological examination are also carried out. Since NOE has potentially life-threatening complications, imaging studies are strongly recommended [3]. Imaging can play synergistic roles in the management of NOE and clinicians must assess both clinical signs and symptoms, radiological imaging and inflammatory markers for desirable disease outcomes. Whilst clinical history, findings on examinations, and microbiology assessment can diagnose the disease accurately, imaging plays an important role in determining the extent of the infection and assessing treatment response. The most commonly used imaging modalities in the diagnosis of NOE are computerized tomography (CT), magnetic resonance imaging (MRI), radionucleotides, and F-18 FDG PET/CT [1].

The review further discusses the advantages of limitations of each imaging modality in detail. 

Challenges

Necrotising otitis externa has historically posed some challenges such as determining the anatomic extent of disease and evaluating management outcomes by clinicians [7]. The availability of the latest imaging technology means that clinicians are now able to use more advanced imaging techniques. Despite the availability of such techniques, a good understanding of the specific strengths and weaknesses of imaging modalities in the diagnosis and follow-up of NOE remains key. 

Given that NOE commonly occurs in those who are immune-compromised or suffering from diabetes, increasing longevity, in turn, leads to increased occurrence of the disease. Therefore, doctors in both primary and secondary health care should be mindful of the presenting symptoms and be able to refer accordingly. Similarly, otolaryngologists or ear, nose and throat (ENT) specialists also need to be aware of the modalities related to timely and accurate diagnosis of the disease, including specific imaging procedures. It is important to note that there is no single imaging modality that can fully address the diagnosis or management of NOE and may require a multi-modal approach that examines both functional anatomical changes [8]. Thus a timely and suitable use of new radiologic modalities and management protocol can lead to improved outcomes which in turn can reduce the need for surgical intervention [1] 

## Review

**Aims and objectives of the systematic review** 

There are a number of issues faced in using appropriate imaging modalities in the timely diagnosis and efficient management of NOE that make this review necessary. As described earlier, there can be a misdiagnosis and delay in timely referrals by doctors in primary health care to otolaryngologists. This can thus result in complications and often surgery required which can compromise the quality of life and also cause strain on health services.

Although there are numerous publications related to NOE, there are very few studies focusing on the role of particular imaging modalities applied at various stages of the disease, starting with diagnosis, management and follow-up. The systematic review thus aims to focus on studies that would not only help understand evolving trends in imaging of NOE over the last three decades but also discuss their impact on the timely diagnosis and better management of the disease. In doing so, the systematic review aimed to highlight if there are any gaps in learning and practice with regards to imaging as a means of diagnosis and management including disease progression. 

Methodology

PRISMA Framework

**Figure 1:**
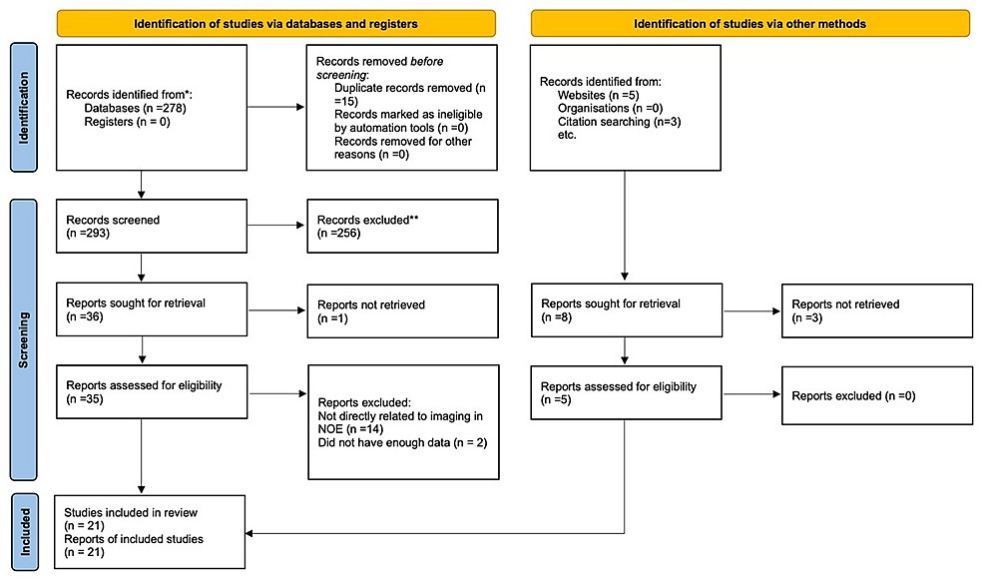
Selection of studies as illustrated by the PRISMA flow chart PRISMA: Preferred Reporting Items for Systematic Reviews and Meta-Analyses; NOE: necrotising otitis externa

A systematic review was carried out using Preferred Reporting Items for Systematic Reviews and Meta-Analyses (PRISMA) 2020 (Figure 1) [9] whereby the author has analysed relevant available studies with regards to various imaging modalities and their appropriate use in diagnosing and managing NOE. Embase, PubMed, Dynamed Plus and National Health Service (NHS) evidence databases were searched. In addition, Cochrane and Google Scholar as well as reference lists were manually checked. Screening, data extraction and quality assessment were undertaken. Data extraction and analysis were cross-checked by two reviewers and a consensus was reached. The author was the main data collector from the studies. Due to the heterogeneous nature of the included studies, a narrative synthesis was eventually performed.

The search strategy applied had extracted data from a wide variety of sources with the use of multiple keywords and thesaurus to ensure that a high number of relevant published studies are revealed. A comprehensive literature review was performed in sources such as PubMed as an interface to search MEDLINE, Research Gate, EMBASE, Wiley Online Library, NHS Evidence Search, Dynamed Plus. Date restrictions were initially set for the last 10 years from 2021. However additional hand search was carried out between 1980 and 2021 to increase the number of relevant studies. In addition to electronic search, a manual search was also applied to ensure the best results are achieved. Cochrane and Google Scholar were also used. The author has also looked into a relevant reference list of related studies to ensure that important papers are not missed out on. The search strategy includes keywords in Medical Subject Heading (MeSH) and Boolean operators "OR" and "AND." Keywords used were radionucleotide scan, indium in-111 labelled leukocyte scanning, gallium citrate ga-67 bone scan, technetium tc-99 bone scan, f-18 fdg pet/ct, ct scan of temporal bone, necrotising external otitis, malignant otitis externa, skull base osteomyelitis. The same keywords were used for an additional hand search. 

MeSH strategy was used as follows: "Diagnostic Imaging"[MeSH] OR "Imaging"[Title/Abstract] OR "CT" [Title/Abstract] OR "Computed Tomography" [Title/Abstract] OR "MRI" [Title/Abstract] OR "Magnetic Resonance Imaging" [Title/Abstract] OR "Scan" [Title/Abstract] Or "Scans"[Title/Abstract] OR "radionucleotide scan" [Title/Abstract] OR "indium in-111 labelled leukocyte scanning" [Title/Abstract] OR "gallium citrate ga-67 bone scan" [Title/Abstract] OR "technetium tc-99 bone scan" [Title/Abstract] OR "f-18 fdg pet/ct," [Title/Abstract] "AND "Skull Base Osteomyelitis" [MeSH] OR " Otitis Externa" [MeSH] AND "necrotizing" [MeSH]OR "necrotising" [MeSH]OR "malignant" [MeSH] AND “last ten years” [PDat] OR “necrotizing otitis externa" [MeSH] OR “necrotising Otitis externa” [MeSH] OR “necrotising external otitis” [MeSH] OR“necrotizing external otitis” [MeSH] OR “malignant otitis externa” [MeSH].

Limitations of the review include the fact that the search yielded very few studies directly related to imaging modalities applied in NOE. However, this was overcome by including studies that looked into the disease in general and discussed the imaging modalities and their impact on diagnosis, treatment and follow-up. There also may be some bias as the systematic review only uses papers in the English language. The author is confident that the search strategy combined with hand searching identified the most relevant studies. Whilst it is still possible that some grey literature was missed, it is unlikely that it would have a major impact on the overall findings. 

Inclusion and Exclusion Criteria

**Figure 2:**
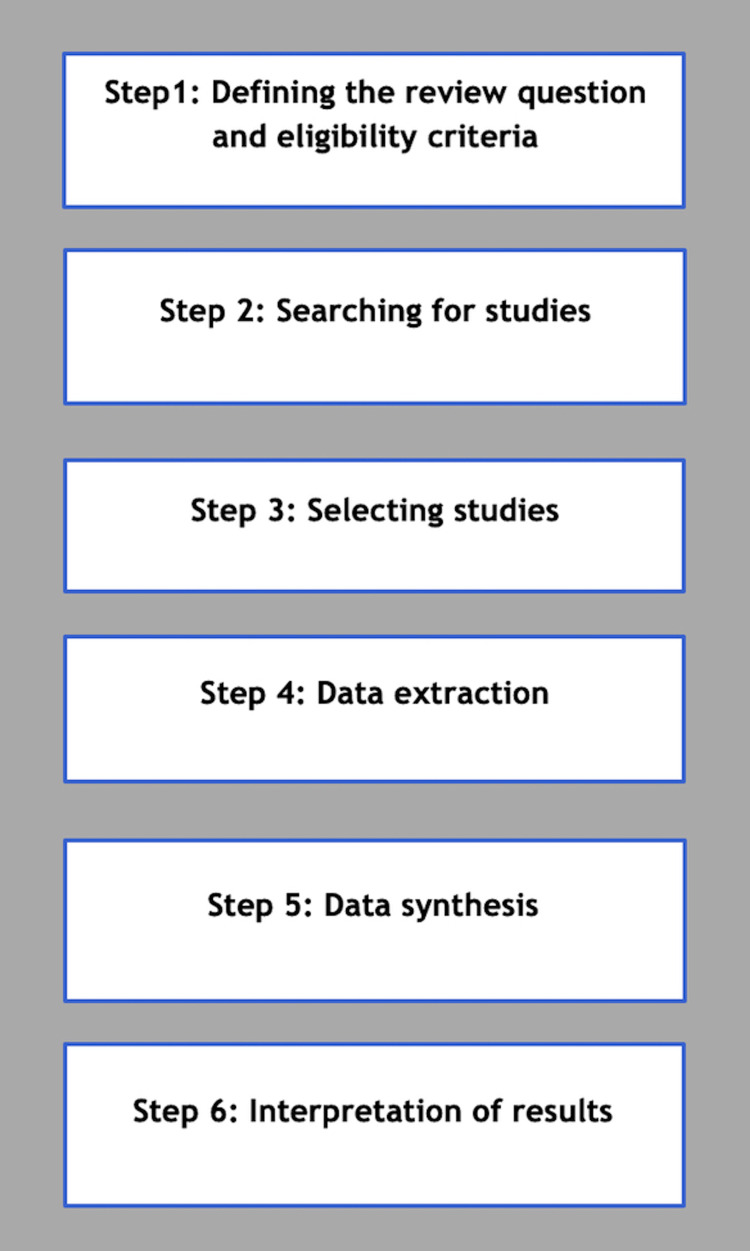
Methodology flow chart

The author reviewed studies related to either diagnosis or management of NOE to analyse various imaging techniques involved. Inclusion and exclusion criteria were established to ensure that the findings of the search yielded more appropriate results. As seen in the flow chart below (Figure 2) this systematic review begins with defining the review question (see aims of the systematic review above) before searching for relevant studies. Data will then be extracted from a number of relevant studies and synthesised to interpret the results and to reach a conclusion. 

The following types of studies were included in our study: publication date: the initial search was from 2008 until 2018 and a second search was made to include publications from 1980 to date; studies from any geographical location; studies in the English language

The following types of participants were included in our study: adults (≥18 years); suspected or diagnosed with NOE and undergoing management such as surgical debridement, long-term intravenous antibiotic therapy with either good or poor outcomes; being treated in the primary, secondary or tertiary care centre;

The following types of studies were excluded from our: non-English language; published pre-1980; studies not published in a peer-reviewed journal; dissertations; proceedings.

Participants below 18 years of age were excluded from our study. 

Risk of bias assessment

There is a risk of publication bias in systematic reviews in general. The author is confident that the search strategy combined with hand searching identified the most relevant studies. Whilst it is still possible that some grey literature was missed, it is unlikely that it would have a major impact on the overall findings. It is also important that systematic reviews comply with established methodology and their findings are free of bias. 

Results

The author reviewed 38 studies. Out of these, 35 studies involved 1204 patients whilst three studies have provided an expert review on the topic. In addition, two studies were surveys that included responses from 221 clinicians in the UK and 136 participants in the U.S. Whilst the statistical significance was not commented on in most studies, the clinical significance was clearly indicated. This characteristic of the studies is thus relevant to the objectives of this article and provides ample evidence to form the basis of any future research in this area.

Some trials compared face-to-face interventions directed at individual patients and the results of these studies constitute higher quality evidence. Out of the 40 studies listed, three studies were conducted in middle-income countries and the remaining 37 were conducted in high-income countries. In addition, the sample sizes of most studies are small and there is limited information available on their follow-up. However, the author analysed 21 studies with relatively more information on imaging techniques used in the diagnosis, management and follow-up of NOE (Table 1). The interventions in these studies comprised a mix of single-session and multi-session strategies. Some of the studies have focused solely on one technique and have not compared it with a control group. This makes it difficult to compare all techniques in the same depth in a particular cohort. In addition, most studies have not examined the use of multiple techniques to validate it. 

**Table 1:** Table of studies related to imaging modalities in diagnosis and follow-up of Necrotising Otis Externa Ga-67: Gallium-67-citrate; Tc99m-MDP: Technetium-99 Methylene Diphosphonate

No	Author, date & country	Patient Group	Study type	Aim of the study	Conclusion
1	Curtin, Wolfe & May 1982, USA [10]	4 cases of Necrotising Otitis Externa (NOE)	Retrospective case study	To analyse the CT scan findings in NOE	CT scan can demonstrate involvement of the soft tissues at the base of the skull well and also correlate well with the clinical findings
2	Parisier SC et al, USA, 1982 [11]	18 patients with NOE between the ages 61-89- average 73 yrs 1(3M 5F)- all with diabetes mellitus (DM)	Retrospective Study	To assess the involvement of temporal bone and base of skull radiologically	Tc99m-MDP bone scan, is a valuable test since results are positive in early cases of osteomyelitis of the temporal bone and base of skull, showed increased uptake in all 18 patients. In 6 patients, Ga-67 citrate scans were obtained at the start of therapy and at 5–6-week intervals thereafter. The serial G67 scans were useful in evaluating the effectiveness of therapy since the uptake decreased with control of infection.
3	Gold S. et al, 1984, USA [12]	23 cases with NOE	Retrospective case	To evaluate radiographic findings and assess whether they were correlated with clinical disease	CT scan can demonstrate the progression of bony disease but cannot be used to follow resolution of central skull base osteomyelitis. Radionuclide scans thus provide better information regarding the overall extent of the inflammatory processes
4	Strashun, Nejatheim & Goldsmith SJ. 1984, USA [13]	10 patients with NOE	Retrospective Study	To compare the findings of initial radiographs, thin-section tomography of temporal bone, CT scans of head and neck, Tc99m-MDP and G-67 scintigraphy, and single-photon emission computed tomography (SPECT) for detection of temporal bone osteomyelitis	Tc99m-MDP and G-67 scintigraphy are more sensitive than radiographs and CT scans for early detection of NOE. Thus, G-67 scintigraphy appears to be more specific for follow-up evaluation of these patients
5	Gherini, Brackmann & Bradley, 1986 USA [14]	4 patients with NOE	Retrospective case review	The aim of the study was to evaluate anatomic extent of soft tissue changes in NOE on various imagining techniques such as MRI, CT, Tc99m-MDP and G-67 scans.	MRI has superior findings to CT, Tc99m-MDP bone scan, and Ga-67 scan in evaluating the anatomic extent of soft tissue changes in NOE
6	Rubin, Curtin & Kamerer , 1990, USA [15]	11 patients with NOE	Retrospective case review	The aim of the study was to evaluate the utility of serial CT scan in management of NOE	Serial CT scans help in early diagnosis and determining the extent of infection and disease progression and resolution
7	Klose & Elies, 1991, West Germany [16]	20 patients with NOE	Prospective Study	To confirm CT scan findings by the intraoperative findings in NOE	CT scans are useful in determining the extension of the disease in the pneumatic system of the skull and the grade of involvement of bones and soft tissues in patients with NOE. A modified classification of NOE based on CT scan findings is proposed
8	Grandis, Curtin & Yu, 1995, USA [17]	7 patients	Prospective study	To compare CT and MR imaging in the diagnosis and follow-up of NOE	CT is preferred at initial diagnosis, as small cortical erosions are better seen. Whilst either modality can be used to follow up soft-tissue evolution, MRI may be superior for evaluation and follow-up of meningeal enhancement and changes within the osseous medullary cavity
9	Stokkel MP et al, 1997, The Netherlands [18]	8 patients (five males, three females) with the clinical diagnosis of NOE	Prospective Study	To establish whether quantitative G-67 single-photon emission tomography (SPET) represents an accurate method for the assessment of infection and monitoring of therapeutic effect in NOE	Imaging techniques such as MRI, CT scan and bone scintigraphy, provide a good picture of the spread of the disease, but the images remain unchanged for a long time even after disease has regressed. It is thus concluded that in addition to the visual analysis of 67Ga SPET imaging, Lesion-to-Non-Lesion (L/NL) ratios should be calculated to get a more accurate picture of the disease progression or resolution in NOE. count. It was also concluded CT scans are of little value for diagnosis or for monitoring of therapeutic effect in such cases
10	Karantanas AH. et al, 2003, Greece [19]	4 patients	Retrospective Case Study	To study the usefulness of CT and MRI in the evaluation of diabetic patients with NEO. The history of the cases, the imaging findings, and the correlation of CT and MRI scans were discussed and analysed	MRI is superior to CT in patients with NOE in terms of estimating the anatomic extent of the disease, but it cannot be used for monitoring therapy
11	Okpala NC et al, 2005, UK [20]	3 cases	Retrospective case review	This study looks at case series of radiological & radionucleotide (anatomical and functional) investigation and discusses their significance in initial diagnosis, management and follow up of NOE	CT and/or MRI should be supplemented by SPECT bone imaging for initial diagnosis of NOE routinely. It also concluded that for follow-up of NOE cases in assessing the response to disease and recurrence, G-67 should be the investigation of choice
12	Sudhoff H. et al, 2008, UK [21]	23 patients (average age 71 years, age range 39-87)	Retrospective analysis	To evaluate usefulness of CT scans in NOE	CT scanning is fast and economical tool in the initial assessment of patients with NOE. CT findings of temporal bone in itself, however, were not closely correlated to the clinical outcome of the patients. Hence limited value in predicting outcome.
13	Al-Noury K &, Lotfy A 2, 2011, Saudi Arabia [22]	18 patients (16 males and 2 females; age, 49-79 years; mean, 65.3 years) with a clinical diagnosis of NOE	Prospective Study	To illustrate the role CT scan and MR-I in the diagnosis and delineation of the extent of disease in NOE at presentation and following successful treatment.	CT and MRI play complementary roles in the diagnosis and follow-up of patients with NOE
14	Chen YH &, Hsieh HJ. 2013,Taiwan [23]	1 case of NOE with initial negative planar scintigraphy finding	Retrospective review	Triphasic bone and 67Ga scintigraphy are used to initially detect and follow-up the response of therapy	Compared with planar image, SPECT/CT image provided better anatomical information and is more sensitive in detecting small lesions. It also concluded that the photon emission CT scan should be performed routinely for patients with suspected NOE, even without evidence of lesion on planar images
15	Cherko, et al. 2016 UK [24]	7 adults diagnosed with NOE, clinically and through CT	Prospective Study	To report on the experience of imaging through Diffusion-Weighted Magnetic Resonance Imaging (DW-MRI) tool for assessing and monitoring treatment response in NOE	DW-MRI investigations were superior to Conventional MRI
16	Adegbiji WA et al. 2017, Nigeria [25]	Data of 9 patients with diagnosis of NOE managed in a tertiary hospital between years 2012 – 2016 was analysed	Prospective Study	To sensitise clinicians for high level of suspicion on early diagnosis and efficient treatment of NOE	Whilst both CT scan & MRI were used CT Scan is the investigation of choice as it delineates subtle changes in bone density and establishes the extent of soft tissue swelling. Radioisotope scans are also useful for monitoring treatment
17	Sharma, Cohrann & Singh, 2017, India [26]	43 patients with NOE	Retrospective review	To assess the impact of the introduction of a dedicated management protocol of NOE patients jointly with departments of otorhinolaryngology and infectious diseases	The introduction of a dedicated management protocol resulted in improved care & decreased length of stay in patients. With regards to imaging it recommends both CT and serial MRI
18	Shamanna K & Ganga VB, 2018 India [27]	30 m & 4 f aged between 48 to 61 years with earache and discharge	Prospective Study	To evaluate the clinical presentation, co-morbid conditions and treatment response in the management of NOE	High-resolution CT (HRCT) scan of temporal bone helps evaluate bone involvement whilst MRI of the head and neck shows soft tissue involvement. This helps assess disease status and also treatment duration. Tc99m-MDP bone scan, Ga-67 bone scan and In-111 labelled leukocyte scanning are useful in detecting bone involvement in the early stages of NOE
19	Cooper, T. et al, 2018, USA [28]	136 participants	Cross-sectional survey study	This was a survey shared amongst neurologists and head and neck radiologists regarding use of imaging in the diagnosis and management of NOE	There is considerable heterogeneity in the preferred imaging modalities used in the diagnosis and management of NOE. CT and MRI are the preferred contemporary modalities used by many physicians, demonstrating a shift away from the historic use of nuclear medicine scans
20	Stern Shavit S. et al, 2019, Israel [29]	12 patients were diagnosed with NOE	Case Series	To suggest 2-deoxy-2-(18F) fluoro-D-glucose positron emission tomography/computed tomography (18F-FDG-PET/CT) as an alternative to Tc-99m MDP and Ga-67 scans for diagnosis and assessment of response to treatment for patients with NOE	18F-FDG-PET/CT is a reliable imaging modality for diagnosis, disease localization, and decision-making regarding treatment cessation of NOE and it should be considered as the imaging modality of choice for initial diagnosis and follow-up in NOE patients. It also concluded that larger, controlled studies are warranted
21	Moss, W. J. et al, 2020, USA&HGK [30]	20 articles with a combined 608 patients	Systematic review and meta-analysis	To assess the sensitivity and specificity of traditional nuclear medicine studies in the diagnosis of NOE	Tc99m-MDP and Ga-67 have poor specificity, lack of anatomic resolution, unproven ability to detect disease resolution in NOE and has variable availability. The review does not support their routine use in the management of NOE

Key Findings of the Review

Most of the studies reviewed highlighted the importance of imagining in addition to physical examination and laboratory testing in the diagnosis as well as prognosis of NOE. Several studies discussed whether one imaging technique is superior to the other, whilst a few studies explored the use of a multi-pronged approach and appropriate use of specific imaging modalities yielding better results in diagnosis, management and disease outcomes. Most studies on NOE have only touched upon imaging briefly and not compared the advantages and limitations in greater detail. 

Given that various clinicians use different techniques, it is important to understand the positive aspects as well as limitations of these techniques and explore a multi-pronged approach. Despite the various gaps and limitations in the studies reviewed, it is evident that each imaging technique used in the diagnosis and management of NOE has its clear advantages and limitations (Table 2). 

**Table 2:** Strength and Weakness of Imaging Modalities in NOE

No	Imaging modality	Strengths	Weaknesses/limitations
1	CT Scan	Useful in the early stages and shows the extent of bone erosion. Economical and widely available.	Little value for diagnosis or for monitoring of therapeutic effect. Radiologic changes on CT only become evident when at least one-third of the bone mineral is lost.
2	MRI	Best for determining the extent of disease in the soft tissue. Method of choice in determining intracranial extension.	Not useful for monitoring therapy.
3	Radionucleotide scans	Useful for the early detection as well as management of the disease.	Poor specificity Lack of anatomic resolution. Variable availability around the world.
4	18F-FDG-PET/CT	Reliable for diagnosis, disease localization, and decision-making regarding treatment cessation of NOE.	Variable availability around the world.

Discussion

The review aimed to look at the advantages and limitations of various imaging modalities in the diagnosis and management of NOE.

Whilst conventional radiology does not have any use in the diagnosis of NOE, the radiological diagnosis of the disease tends to remain limited largely to computed tomography (CT) scans and magnetic resonance imaging (MRI) and are often used in complementarity [16]. A study recommended the use of both high-resolution CT temporal bones to define bony erosion and serial MRI with contrast to monitor treatment response [17]. 

Whilst CT and MRI are used for anatomical imaging, nuclear techniques are useful in understanding the functional process [13], depending on the availability of resources, Technetium-99 Methylene Diphosphonate (Tc-99m MDP) bone scanning, Gallium-67-citrate (Ga-67) bone scanning and Indium 111 labelled leukocyte scanning were also widely used according to some studies [8]. However, the review notes their current usage as newer and more effective imaging modalities have become available, such as 2-deoxy-2-(18F) Fluoro-D-Glucose Positron Emission Tomography/CT (18F-FDG-PET/CT) [29].

Studies showed that CT scanning is a fast and economical tool in the initial assessment of patients with NOE [18]. It allows determining the location and extent of disease, whilst MRI is the method of choice in determining intracranial extension [19] [20]. While CT scanning allows determining the location and extent of disease, MRI is the method of choice in determining intracranial extension. Some studies concluded that serial CT scans help in early diagnosis, and determine the extent of infection and disease progression and resolution [21]. 

A limitation for both CT scan and MRI is that active inflammation and resolving infection is hard to differentiate and hence CT and MRI imaging does not necessarily correlate to the clinical outcome of the disease. However, bone scintigraphy can detect NOE earlier than any CT or MRI and hence these should be accompanied for the initial diagnosis [21]. The advantage of CT scanning over other modalities is detecting bony erosions and demineralization [23]. The review also found studies that concluded that MRI is superior to CT scan [24]. A study concluded that diffusion-weighted MRI (DW-MRI) investigations were superior to conventional MRI [25]. An earlier study suggested that MRI has superior findings to CT, Tc-99 bone scan, and Ga-67 citrate scan in evaluating the anatomic extent of soft tissue changes in NOE [25]. MRI is thus regarded as the imaging technique of choice [14]. MRI can also demonstrate meningeal enhancement better and can reveal the intracranial extension of the disease as well and reveal complications such as thrombosis and intracranial spread.

Some studies showed the usefulness of that Ga-67 Tc-99m MDP bone scanning and concluded that these are more sensitive than radiographs and CT scans for early detection of NOE and hence both are important imaging tools in diagnosis as well as management of the disease. [21] [22]. Whilst Tc-99m MDP can be useful for the initial evaluation of the disease, is not useful in assessing the progress of the disease. Tc-99m MDP stays positive for a long period, even after the resolution of the infection [2] [9]. Ga-67 is noted as a useful tool to monitor the resolution of the disease which can be seen as decreased uptake in the affected area [2]. However, to get a more accurate picture, the lesion to non-lesion ratio needs to be determined [18]. Indium In 111-labelled leukocyte scans on the other hand show similar findings as a Ga-67 scan, it is more specific to an inflammatory process. It is also reliable and a timely indicator of the resolution of infection [9].

It is also worth noting that 18F-FDG-PET/CT is described as a reliable imaging modality for diagnosis, disease localization, and decision-making regarding treatment cessation of NOE [29] and considers this as the imaging modality of choice for initial diagnosis and follow-up in NOE patients. Some earlier, studies also showed that CT and/or MRI can be supplemented by single-photon emission computed tomography (SPECT) bone imaging for the initial diagnosis of NOE routinely as well as for follow-up of NOE cases. Whilst SPECT can also be useful in assessing the response to disease and recurrence, Ga-67 is being referred to be the investigation of choice to assess disease progression [20]. However, more recent studies do not favour Tc99m-MDP and Ga-67 and conclude that these have poor specificity, lack anatomic resolution and the ability to detect disease resolution in NOE is unproven. In addition, both have variable availability and thus their routine use in the management of NOE is not recommended [30].

From this review, it is evident that clinicians have varied approaches to imaging modalities of NOE.

As mentioned earlier, diagnosis of NOE requires a high suspicion index. Thus, lack of clarity on initial examination and disproportionate symptoms to clinical findings can further delay diagnosis. Often disease progression remains unclear until radiological findings and radio-nucleotide results are obtained. It is therefore imperative that a sound radiological assessment should be supplemented by clinical and serological analysis to establish the disease [1] and avoid delay in diagnosis. Hence sound knowledge of the use of particular imaging techniques at various stages of disease - from initial stages of identification, disease progression and resolution - will lead to better management outcomes and reduced need for surgical intervention [15]. The studies highlight that there may not be one imaging technique that is superior to the other but it is the appropriate selection and timely use of each modality that can have a positive impact on disease outcomes. Whilst most of these studies have not ideally demonstrated the use of multiple modalities in detail, some do discuss the strengths and weaknesses of each technique, which forms the basis of further research in exploring a multi-pronged approach to imaging. 

The studies reviewed also demonstrate that imaging in NOE has not changed drastically over the years but some newer and more effective modalities techniques are being preferred depending on the availability of resources. In addition, whilst clinical presentation, diagnosis, and pathophysiology of NOE are well understood, there is still no consensus on the best use of imaging techniques [13]. Various imaging modalities have their own advantages and limitations which need to be fully understood to achieve optimal management outcomes. The availability of the latest imaging technology means that clinicians are now able to use more advanced imaging techniques in many parts of the world. Despite the availability of such techniques, NOE continues to pose some challenges such as appropriate imaging modalities in diagnosis and throughout disease progression and follow-up due to its complex nature. 

Implications for practice and further research

Whilst the studies reviewed examined most of the important outcomes of the intended research, there is still limited research available with regards to consensus on the use of imaging methodologies in NOE in detail. Hence there is clearly a gap in this particular area. Moreover, many studies provide limited information on how they concluded their findings and do not discuss the use of a multi-pronged approach in order to achieve positive management outcomes. In addition, the availability or lack thereof with regards to imaging modalities and the challenges it may pose for timely detection and appropriate management and follow-up by some clinicians, especially in less developed settings is an area that requires further research. Overall there is a need to improve the methodological and statistical quality of studies and the use of objective and validated outcome measures related to this very specific area of clinical practice.

## Conclusions

This systematic review looked at studies that highlight the strengths and weaknesses of imaging techniques related to NOE and the significance of each technique at various stages of disease such as diagnosis, progression and resolution. The review notes that imaging in NOE has not changed drastically over the years but some newer and more effective modalities techniques are being preferred depending on the availability of resources. In addition, various imaging modalities have their own strengths and weaknesses. For example, as various studies establish, a CT scan is useful in the early stages and shows the extent of bone erosion and demonstrates the progression of the bony disease. However, it cannot be used to follow resolution in cases where there is a possible central skull base osteomyelitis or analyse the impact of treatment. Similarly, MRI is superior to CT scan in detecting anatomical locations and since it has a superior contrast resolution, whilst radionuclide scans can provide better information on the overall spread of inflammation. Hence, no single imaging modality can fully address the scope of NOE. Whilst clinicians have increased reliance on CT scans and MRI in the initial diagnosis and some aspects of follow-up of NOE, a combination of modalities and their stage-specific use remains critical for positive disease outcomes and there continues to be no "gold standard" for establishing disease resolution.
